# Prognosis of patients with severe hyponatraemia is related not only to hyponatraemia but also to comorbidities and to medical management: results of an observational retrospective study

**DOI:** 10.1186/s12882-016-0370-z

**Published:** 2016-10-22

**Authors:** Thierry Krummel, Eric Prinz, Marie-Astrid Metten, Claire Borni-Duval, Dorothée Bazin-Kara, Emmanuelle Charlin, Jean-Marc Lessinger, Thierry Hannedouche

**Affiliations:** 1 Department of Nephrology and Dialysis, University Hospital, Strasbourg, France; 2 Department of Biostatistics, University Hospital, Strasbourg, France; 3 Department of Nephrology, General Hospital of Colmar, Colmar, France; 4 Laboratory of Biochemistry and Molecular Biology, University Hospital, Strasbourg, France; 5 School of Medicine, University of Strasbourg, Strasbourg, France

**Keywords:** Clinical decision-making, Hyponatraemia, Patient care management, Prognosis, Retrospective studies

## Abstract

**Background:**

The true cause of death in severe hyponatraemic patients remains controversial. The present study aimed to analyse the relationship between comorbidity, medical management and prognosis in severe hyponatraemic patients.

**Methods:**

Medical records of all patients hospitalised in our institution in 2012 with a plasma sodium ≤120 mmol/l were retrospectively analysed.

**Results:**

One hundred forty-seven of 64 723 adult patients (0.2 %) were identified with severe hyponatraemia. In-hospital mortality rate was 24.5 and 50.3 % after a median follow-up of 431 days. Patients with plasma sodium <110 mmol/l had less comorbidity (Charlson Comorbidity Index 2.2 ± 1.9 vs. 4.0 ± 3.1 (plasma sodium 110–115 mmol/l) and 4.2 ± 3.1 (plasma sodium 116–120 mmol/l); *P* = .02)) and a small trend for less mortality, respectively 40.0, 51.2 and 52.3 % (*P* = .64). At discharge, nonsurvivors and survivors had similar plasma sodium with 58.3 % of nonsurvivors being normonatraemic. Urine analysis was performed in 74.2 % of cases and associated with lower in-hospital mortality (20.2 % vs. 36.8 %, *P* = .05). In multivariate Cox analysis, mortality was significantly associated with plasma sodium normalisation (HR 0.35, *P* < 0.001), urine analysis (HR 0.48, *P* = .01), Charlson Comorbidity Index (HR 1.23, *P* < .001) and serum albumin (HR 0.88, *P* < .001).

**Conclusion:**

Mortality in severe hyponatraemia appears mainly due to comorbidities although the latter are potentiated by hyponatraemia itself and its management thereby exacerbating the risk of death.

## Background

Hyponatraemia is the most common electrolyte disorder observed in hospitalised patients and is closely associated with in-hospital mortality even in mild cases [1–9]. The high mortality rate associated with severe hyponatraemia suggests a causal relationship [4, 10–12]. However, the relationship between the magnitude of hyponatraemia and mortality remains a matter of debate and the disease causing hyponatraemia may be more responsible for the observed mortality than hyponatraemia per se [13–15].

Severe hyponatraemia is symptomatic in nearly half of patients [10, 13, 16], while neurologic symptoms secondary to cerebral oedema require urgent therapeutic care, guided by a rigorous diagnostic approach. However, quality of care is often inadequate [17–20], which can further contribute to mortality [21, 22].

The present study, conducted in a cohort of patients with severe hyponatraemia in our institution, aimed to analyse the short- and medium-term prognosis, the factors associated with mortality, as well as the diagnostic and therapeutic management of these patients. The main objective was to analyse the medical care delivered and its relationship with the prognosis, while the secondary objectives were to examine whether prognosis was influenced by the severity of the hyponatraemia and to analyse the link between severity of hyponatraemia and comorbidity.

## Methods

### Patient recruitment

**Fig. 1:**
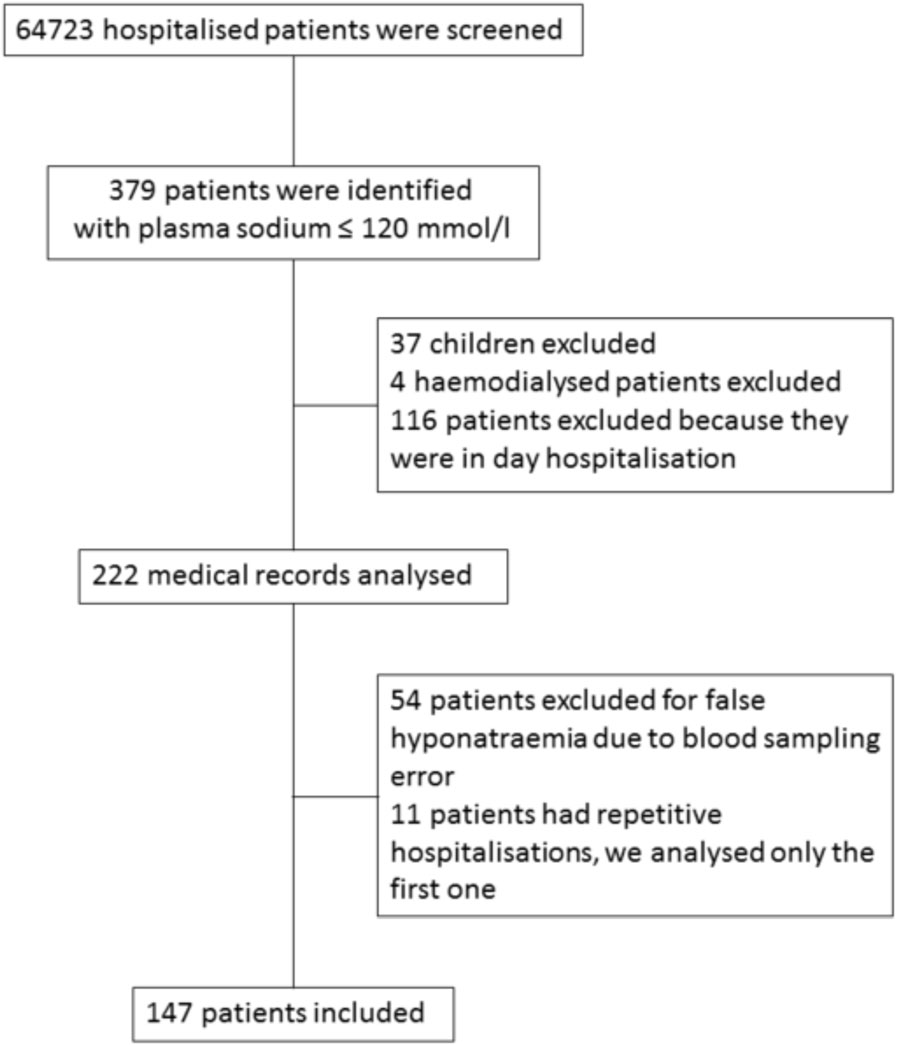
Flow chart

Using computer retrieval of archived laboratory data, all adult patients with a measured plasma sodium of less than or equal to 120 mmol/l during a hospitalisation at the University Hospital of Strasbourg from January 1^st^ 2012 to December 31^st^ 2012 were identified. Among 64 723 patients hospitalised, 379 were identified with severe hyponatraemia of which 147 adults were retained and included in the retrospective study (Fig. 1). Excluded patients were children, ambulatory hospital patients, repeat hospitalisations, patients in which blood samples were obviously diluted or patients with an inaccessible medical record. Patients were divided into 3 groups according to their nadir plasma sodium: group A: <110 mmol/l; group B: 110–115 mmol/l; group C: 116–120 mmol/l.

### Collected data

Data were collected through a retrospective analysis of medical records and included the following items: department of hospitalisation, patient demographics, associated comorbidities entering in the calculation of the prognostic Charlson comorbidity index, aetiological workup and treatment modalities of hyponatraemia, final aetiological diagnosis, time course of plasma sodium and other pertinent biological data (osmolality, plasma glucose, serum creatinine and estimated glomerular filtrations rate (eGFR), thyroid and adrenal hormones, serum protein, albumin and Brain Natriuretic Peptide (BNP)). Hyponatraemia was classified as either acute or chronic if developed in less or more than 48 h respectively, and either as community-acquired if present on admission or hospital-acquired. Date of death was also recorded if the patient had deceased. When surviving patients were discharged from hospital, the administrative record of the city of birth was enquired for outcome. The follow-up was established from the day of the first plasma sodium below or equal to 120 mmol/l and the last outcome assessment date of February 15th, 2014.

Plasma sodium was measured by indirect potentiometry (ADVIA2400, Siemens, Germany) while plasma albumin was measured with the bromocresol green method at the same laboratory.

### Statistical analysis

Quantitative variables are presented as mean ± standard deviation (SD) and categorical variables as percentage for each modality.

After analysis of the distribution of variables with the Shapiro-Wilk test, means were compared by a parametric test (Student’s test or ANOVA if more than two groups) or non-parametric test (Wilcoxon rank-sum test or Kruskal-Wallis if more than two groups). Correlations were tested with the Pearson test if at least one of the variables was normally distributed and with the Spearman test in other instances. Categorical variables were compared with Fisher’s exact test. Survival curves were computed according to the Kaplan-Meier method and compared with the Log-Rank test.

Univariate and multivariate analysis of mortality factors was performed using a Cox model. Multivariate analysis included relevant parameters and those with a P-value less than 0.2 in univariate analysis. Multivariate analysis was completed by an interactions search and by a proportionality test.

All statistical analyses were performed using STATA 13.1 software (Stata Inc, Texas, USA), with *P* < 0.05 in 2-tailed tests considered statistically significant.

## Results

### Population characteristics

The characteristics of the 147 included patients are listed in Table 1. Mean and median follow-up was 347 ± 273 days and 431 days respectively, ranging from 1 to 766 days [IQ 34–587].

**Table 1:** Patient characteristics

		All patients (*n* = 147)	Group A (*n* = 20)	Group B (*n* = 41)	Group C (*n* = 86)	*P* ^a^
Age (years)	*n* = 147	69.6 ± 13.3	68.8 ± 14.5	69.5 ± 13.2	69.9 ± 13.3	.95
Males/Females (%)	*n* = 147	42.2/57.8	35.0/65.0	48.8/51.2	40.7/59.3	.57
Weight (kg)	*n* = 116	64.6 ± 16.5	62.3 ± 14.6	67.8 ± 18.0	63.4 ± 16.1	.39
BMI (kg/m^2^)	*n* = 79	24.1 ± 5.8	22.0 ± 5.1	25.2 ± 7.2	24.0 ± 5.1	.37
Plasma sodium at admission (mmol/l)	*n* = 147	121 ± 10.4	110 ± 11.7	119 ± 8.4	126 ± 8.5	<.001
Nadir plasma sodium (mmol/l)	*n* = 147	115 ± 4.7	105 ± 3.4	113 ± 1.4	118 ± 1.0	<.001
Measured plasma osmolality (mosm/l)	*n* = 50	254 ± 18	240 ± 13	252 ± 12	265 ± 18	<.001
Plasma sodium at discharge (mmol/l)						
- Survivors - Nonsurvivors	*n* = 111 *n* = 36	132 ± 6.5 132 ± 11.2	133 ± 8.6 124 ± 18.4	134 ± 8.9 133 ± 13.8	131 ± 7.2 132 ± 9.1	.24 .61
Delta plasma sodium (mmol/l) ^b^	*n* = 147	20.8 ± 8.6	31.1 ± 6.5	23.0 ± 8.2	17.4 ± 6.9	<.001
Serum potassium (mmol/l)	*n* = 147	4.2 ± 1.0	3.8 ± 1.0	4.0 ± 0.9	4.4 ± 1.0	.01
Urea (mmol/l)	*n* = 147	10.1 ± 10.4	7.0 ± 9.6	8.4 ± 9.1	11.7 ± 10.9	.02
Plasma creatinine (μmol/l)	*n* = 147	118 ± 134	83 ± 115	93 ± 84	138 ± 153	.03
eGFR (ml/min/1.73 m^2^)	*n* = 147	100 ± 65.1	131 ± 65.3	100 ± 58.7	93 ± 66.5	.05
Uric acid (μmol/l)	*n* = 59	336 ± 237	258 ± 261	285 ± 246	386 ± 220	.04
Plasma glucose (g/l)	*n* = 143	1.54 ± 1.16	1.50 ± 0.58	1.32 ± 0.90	1.66 ± 1.35	.08
Triglycerides (g/l)	*n* = 40	0.83 ± 0.33	0.73 ± 0.30	0.88 ± 0.30	0.85 ± 0.40	.51
Total protein (g/l)	*n* = 100	62 ± 8.3	62 ± 6.1	63 ± 9.3	62 ± 8.4	.89
Serum albumin (g/l)	*n* = 110	34 ± 6.0	36 ± 3.9	35 ± 5.7	33 ± 6.6	.19
TSH	*n* = 72	3.66 ± 9.1	1.71 ± 2.58	3.73 ± 10.31	4.47 ± 10.2	.03
Plasma cortisol	*n* = 26	230 ± 106	247 ± 95	191 ± 75	240 ± 122	.59
BNP	*n* = 55	453 ± 586	537 ± 331	382 ± 545	472 ± 646	.27

Group A: plasma sodium <110 mmol/l; group B: plasma sodium 110–115 mmol/l; group C: plasma sodium 116–120 mmol/l. *BMI* Body Mass Index, *eGFR* estimated Glomerular Filtration Rate, *TSH* Thyroid Stimulating Hormone, *BNP* Brain Natriuretic Peptide. ^a^between group A, B and C. ^b^Delta plasma sodium is the difference between the highest and the lowest plasma sodium during hospitalisation

Age, sex ratio, weight and body mass index did not differ between the 3 levels of hyponatraemia. However, patients with the lowest plasma sodium also had the lowest plasma potassium, urea, creatinine and uric acid. The eGFR was higher in patients with the lowest plasma sodium although did not reach statistical significance. Triglycerides, plasma glucose and cortisol, BNP, total serum protein and albumin were comparable across all three levels of hyponatraemia whereas thyroid-stimulating hormone (TSH) appeared inversely correlated with plasma sodium. The latter was linked to the presence of 3 patients with very high TSH values, 1 in the intermediate group and 2 in the higher plasma sodium group.

### Comorbidities

**Fig. 2:**
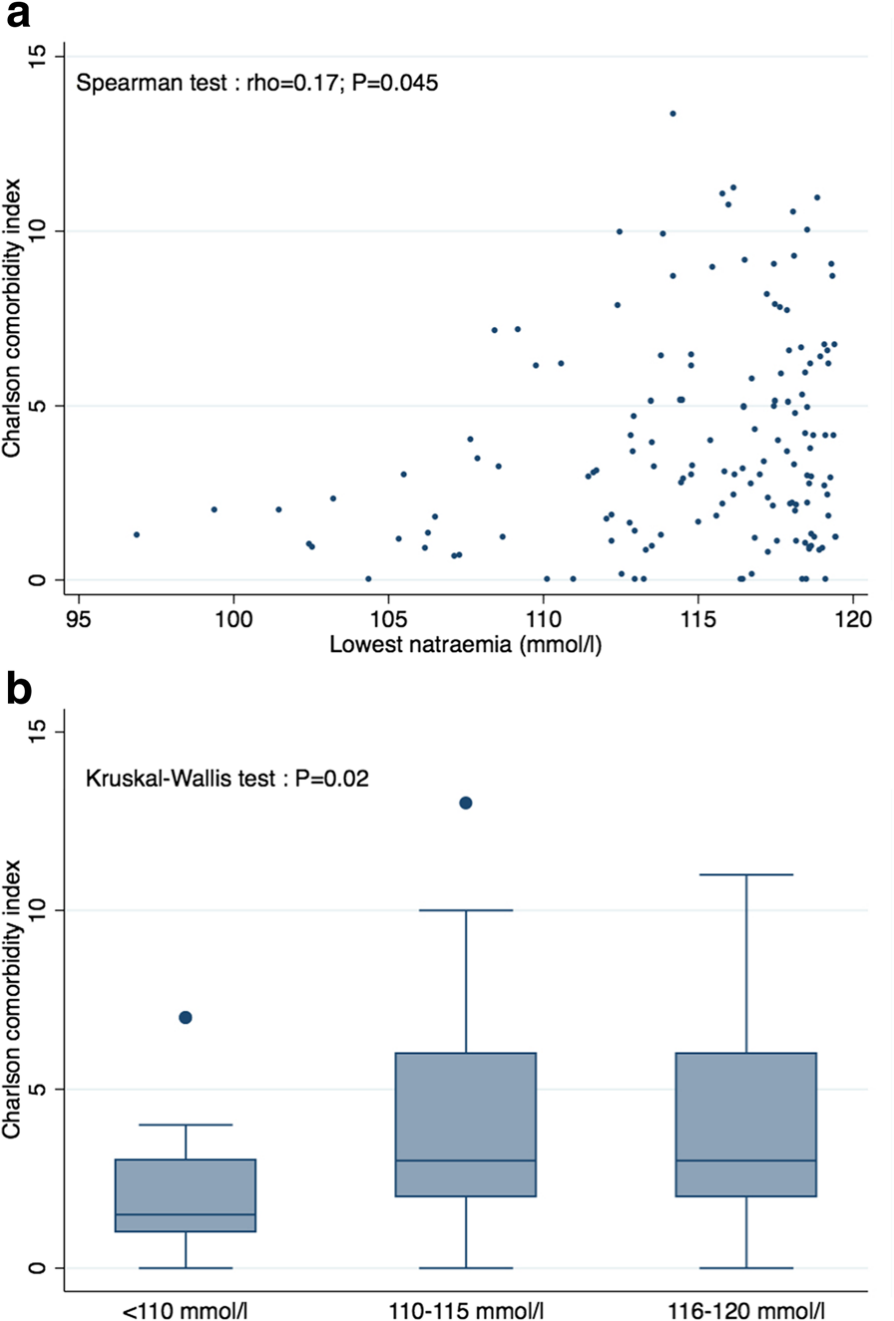
Relationship between plasma sodium and the Charlson comorbidity index. **a** scatterplot of the Charlson comorbidity index according to the nadir plasma sodium. **b** boxplot of the Charlson comorbidity index according to different groups of nadir plasma sodium, <110 mmol/l, 110 to 115 mmol/l and 116 to 120 mmol/l

Patients with the most severe hyponatraemia had less comorbidities along with a Charlson comorbidity index significantly lower than patients in the intermediate and higher plasma sodium groups, with a significant correlation observed between the Charlson index and the nadir of plasma sodium (Table 2; Fig. 2). Aside from central neurologic diseases such as dementia and stroke which were more frequent in patients with the lowest plasma sodium, the other comorbidities were more frequent in the intermediate and higher plasma sodium groups, especially for cardiovascular, lung and kidney diseases (Table 2). However, between-group difference was only statistically significant for dementia.

**Table 2:** Patient comorbidities according to severity of hyponatraemia

	All patients (*n* = 147)	Group A (*n* = 20)	Group B (*n* = 41)	Group C (*n* = 86)	*P* ^a^
Department of hospitalisation					
- Medicine (%) - Surgery (%) - Intensive care (%)	105 (72.9) 14 (9.7) 25 (17.4)	13 (68.4) 0 (0) 6 (31.6)	31 (79.5) 1 (2.6) 7 (18.0)	61 (70.9) 13 (15.1) 12 (14.0)	.05
Charlson comorbidity index	3.8 ± 3.0	2.2 ± 1.9	4.0 ± 3.1	4.2 ± 3.1	.02
Myocardial Infarction (%)	31 (21.1)	2 (10.0)	11 (26.8)	18 (20.1)	.34
Heart failure (%)	29 (19.7)	2 (10.0)	8 (19.5)	19 (22.1)	.53
Peripheral artery disease (%)	23 (15.7)	0 (0)	9 (22.0)	14 (16.3)	.07
Stroke (%)	18 (12.2)	4 (20.0)	3 (7.3)	11 (12.8)	.34
Dementia (%)	9 (6.1)	4 (20.0)	1 (2.4)	4 (4.7)	.03
Chronic lung disease (%)	31 (21.1)	2 (10.0)	13 (31.7)	16 (18.6)	.12
Chronic liver disease (%)	12 (8.2)	1 (5.0)	1 (2.4)	10 (11.6)	.21
Diabetes (%)	33 (22.5)	3 (15.0)	8 (19.5)	22 (25.6)	.57
Chronic kidney disease (%)	23 (15.7)	0 (0)	7 (17.1)	16 (18.6)	.10
Cancer (%)	57 (38.8)	6 (30.0)	19 (46.3)	32 (37.2)	.43

Group A: plasma sodium <110 mmol/l; group B: plasma sodium 110–115 mmol/l; group C: plasma sodium 116–120 mmol/l. ^a^between group A, B and C

### Symptoms

In the entire cohort, 56.5 % of patients had symptoms related to hyponatraemia, among whom 8.8 % had severe neurologic symptoms including seizures and coma (Table 3). Almost all patients in the lowest plasma sodium group were symptomatic while one third had severe neurologic symptoms. In the intermediate and higher plasma sodium groups, approximately 50 % of patients were symptomatic although less than 5 % presented severe neurologic symptoms. The frequency of confusion, digestive symptoms, unsteadiness and other symptoms was not significantly different between the lower, intermediate and higher plasma sodium groups.

**Table 3:** Hyponatraemia associated symptoms

		All patients (*n* = 147)	Group A (*n* = 20)	Group B (*n* = 41)	Group C (*n* = 86)	*P* ^a^
Patients with symptoms (%)	*n* = 147	83 (56.5)	19 (95.0)	23 (56.1)	41 (47.7)	<.001
Severe neurologic symptoms (%)	*n* = 147	13 (8.8)	7 (35.0)	2 (4.9)	4 (4.7)	.001
- Seizure (%)	*n* = 147	9 (6.1)	4 (20.0)	1 (2.4)	4 (4.7)	.03
- Coma (%)	*n* = 147	4 (15.0)	3 (15.0)	1 (2.4)	0 (0)	.003
Confusion (%)	*n* = 147	42 (28.6)	10 (50.0)	11 (26.8)	21 (24.4)	.08
Nausea/vomiting (%)	*n* = 147	17 (11.6)	3 (15.0)	6 (14.6)	8 (9.3)	.50
Gait disturbance/Fall (%)	*n* = 147	20 (13.6)	4 (20.0)	7 (17.1)	9 (10.5)	.38
Other symptoms (%)	*n* = 147	7 (4.8)	2 (10.0)	2 (4.9)	3 (3.5)	.32

Group A: plasma sodium <110 mmol/l; group B: plasma sodium 110–115 mmol/l; group C: plasma sodium 116–120 mmol/l. ^a^between group A, B and C

### Acute vs. chronic severe hyponatraemia

Acute severe hyponatraemia occurred in 26 patients (17.7 %) while severe hyponatraemia was chronic in only 10 patients (6.8 %) (Table 4). Severe hyponatraemia resulted from an acute aggravation of chronic hyponatraemia in 28 patients (19.1 %) while timing was unclassifiable in 83 patients (56.5 %). Seventy-five percent of patients with the most severe hyponatraemia could not be classified. Severe neurologic symptoms were present in 11.5, 3.6, 10.0 and 9.6 % respectively for acute, acute-on-chronic, chronic and unclassified severe hyponatraemia (*P* = .70). In-hospital mortality appeared higher in acute cases although without statistical significance: 30.8, 39.3, 20.0 and 18.1 % in acute, acute-on-chronic, chronic and unclassified severe hyponatraemia respectively (*P* = .11).

**Table 4:** Timing of hyponatraemia according to severity of hyponatraemia

	All patients (*n* = 147)	Group A (*n* = 20)	Group B (*n* = 41)	Group C (*n* = 86)	*P* ^a^
Timing					
- Present at admission (%) - Acquired in hospital (%)	95 (64.6) 52 (35.4)	16 (80.0) 4 (20.0)	31 (75.6) 10 (24.4)	48 (55.8) 38 (44.2)	.03
Timing					
- Acute (%) - Acute-on-chronic (%) - Chronic (%) - Unknown (%)	26 (17.7) 28 (19.1) 10 (6.8) 83 (56.5)	4 (20.0) 0 (0) 1 (5.0) 15 (75.0)	5 (12.2) 6 (14.6) 3 (7.3) 27 (65.9)	17 (19.8) 22 (25.6) 6 (7.0) 41 (47.7)	.07

Group A: plasma sodium <110 mmol/l; group B: plasma sodium 110–115 mmol/l; group C: plasma sodium 116–120 mmol/l. ^a^between group A, B and C

### Community-acquired vs. hospital-acquired severe hyponatraemia

Severe hyponatraemia was acquired in hospital in 52 patients (35.4 %) and present on admission, i.e. community-acquired, in 95 patients (64.6 %) (Table 4). The proportion of community-acquired hyponatraemia increased with the severity of hyponatraemia (Table 4) and community-acquired hyponatraemia was mildly more severe (Table 6). The frequency of severe neurologic symptoms did not differ between the 2 groups.

Patients with hospital-acquired severe hyponatraemia exhibited a higher Charlson index and a greater number of potential causes (Table 6). While no aetiological diagnosis for the hyponatraemia was reported in medical records in nearly 50 % of cases, the most frequently mentioned diagnoses were the syndrome of inappropriate antidiuretic hormone secretion (SIADH) in 19.1 % of cases and dehydration in 15 % of cases. Although not statistically significant, the lack of diagnosis was more frequent in hospital-acquired severe hyponatraemia whereas SIADH was mentioned twice as frequently in community-acquired cases than in hospital-acquired cases. Similarly, the absence of diagnosis was marginally less frequent in milder hyponatraemia (54.7 % in the 116–120 mmol/l group; 39.0 % in the 110–115 mmol/l group; 30.0 % in the <110 mmol/l group; *P* = .07).

### Aetiological workup

Urine analysis was performed in only 75 % of cases (Table 6), particularly for measuring urinary sodium. Urine urea was measured in approximately the same proportion of cases whereas urine osmolality and urine uric acid were measured in only 25.9 % and 11.6 % of cases, respectively. Urine analysis was performed in the same proportion of cases in hospital-acquired and community-acquired severe hyponatraemia. In contrast, urine analysis was prescribed marginally more frequently with increasing severity of hyponatraemia (67.4 % in the 116–120 mmol/l group; 80.5 % in the 110–115 mmol/l group; 90.0 % in the <110 mmol/l group) (*P* = .07). Although normalisation of plasma sodium was not more frequent when urine electrolytes were measured (55.8 % vs. 55.3 %, *P* = 0.85), measurement of urine electrolytes was associated with a trend in reduced in-hospital mortality (20.2 % vs. 36.8 %, *P* = .05) and overall mortality (46.8 % vs. 60.5 %, *P* = .19).

### Diagnosis

Overall, in 46.9 % of patients, no etiological diagnosis for the hyponatraemia was found in the medical record. Although not statistically significant, this proportion was found to decrease with the severity of the hyponatraemia (Table 5). Among the different causes of hyponatraemia, the increase in the proportion of SIADH in the more severe cases should be highlighted. The presence of an aetiological diagnosis in the medical record was much more frequent when urine analysis was performed (64.2 % vs. 21.1 %, *P* < 0.001).

**Table 5:** Diagnosis, treatments and outcomes according to severity of hyponatraemia

	All patients (*n* = 147)	Group A (*n* = 20)	Group B (*n* = 41)	Group C (*n* = 86)	*P* ^a^
Diagnosis					
Unknown (%)	69 (46.9)	6 (30.0)	16 (39.0)	47 (54.7)	.07
Potomania (%)	8 (5.4)	3 (15.0)	3 (7.3)	2 (2.3)	.04
Heart failure (%)	13 (8.8)	1 (5.0)	2 (4.9)	10 (11.6)	.37
Cirrhosis (%)	4 (2.7)	0 (0)	1 (2.4)	3 (3.5)	.68
Diuretics (%)	6 (4.1)	1 (5.0)	1 (2.4)	4 (4.7)	.82
Hypotonic fluids (%)	3 (2.0)	0 (0)	1 (2.4)	2 (2.3)	1.0
Dehydration (%)	22 (15.0)	4 (20.0)	7 (17.1)	11 (12.8)	.61
CSWS (%)	2 (1.4)	0 (0)	1 (2.4)	1 (1.2)	.66
SIADH (%)	28 (19.1)	6 (30.0)	12 (29.3)	10 (11.6)	.03
Hypothyroidism (%)	3 (2.0)	2 (10.0)	1 (2.4)	0 (0)	.02
Adrenal insufficiency (%)	4 (2.7)	1 (5.0)	0 (0)	3 (3.5)	.42
Treatment (%)	127 (86.4)	20 (100)	37 (90.2)	70 (81.4)	.06
- Fluid restriction (%) - Cause withdrawal (%) - Isotonic saline (%) - Hypertonic saline (%) - Furosemide (%) - Demeclocycline (%)	93 (63.3) 31 (21.1) 67 (45.6) 16 (10.9) 19 (12.9) 10 (6.8)	17 (85.0) 8 (40.0) 8 (40.0) 9 (45.0) 2 (10.0) 4 (20.0)	28 (68.3) 9 (22.0) 22 (53.7) 2 (4.9) 6 (14.6) 4 (9.8)	48 (55.8) 14 (16.3) 37 (43.0) 5 (5.8) 11 (12.8) 2 (2.3)	.04 .07 .47 <.001 .94 .01
Plasma sodium normalisation (%)	84 (57.1)	12 (60.0)	24 (58.5)	48 (55.8)	.92
Death during hospitalisation (%)	36 (24.5)	2 (10.0)	13 (31.7)	21 (24.4)	.17
Death during follow-up (%)	74 (50.3)	8 (40.0)	21 (51.2)	45 (52.3)	.64

Group A: plasma sodium <110 mmol/l; group B: plasma sodium 110–115 mmol/l; group C: plasma sodium 116–120 mmol/l. *CSWS* Cerebral Salt Wasting Syndrome, *SIADH* Syndrome of Inappropriate Anti-Diuretic Hormone secretion. ^a^between group A, B and C

### Treatment

No specific treatment was prescribed in 13.6 % of patients while fluid restriction was instituted in 63.3 %, isotonic saline in 45.6 % and hypertonic saline in 10.9 % of patients (Table 5). The treatments known to be more specific for hyponatraemia such as fluid restriction, hypertonic saline and demeclocycline were more frequently used in the more severe group. Tolvaptan was not used because not marketed in France at this time. Untreated patients had numerous comorbidities with a Charlson index score of 5.5 ± 0.7 vs. 3.6 ± 0.3 (*P* = .01) as well as a high mortality rate (80 % vs. 45.7 %, *P* = .007).

Plasma sodium was normalised in 57.1 % of patients and the proportion of plasma sodium normalisation was similar in the 3 groups of hyponatraemia severity.

In 26 (18.1 %) patients, the correction rate was considered to be excessive (≥12 mmol/L at 24 h or ≥18 mmol/L at 48 h). Among the latter, mortality did not differ, whether during hospitalisation (19.2 % vs. 24.6 %, *P* = .80) or on the long term (38.5 % vs. 52.5 %, *P* = .28). No biological control was carried out within the 6 first hours following initiation of treatment in 83.0 % of treated patients and within the first 24 h in 17.7 % of treated patients. No osmotic demyelination syndrome was mentioned.

### Mortality

**Fig. 3:**
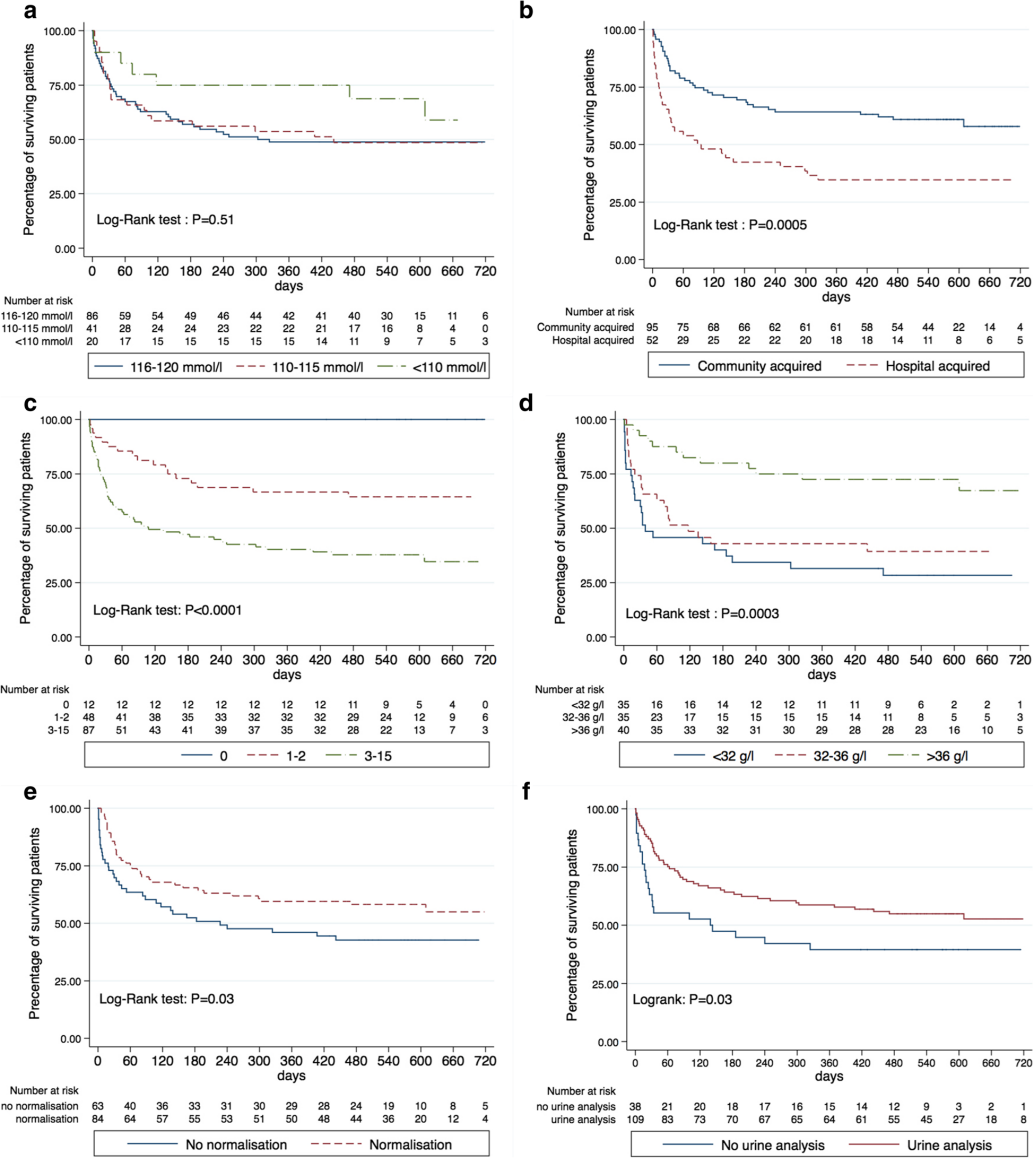
Patient survival curves. Survival according to: **a** the nadir of plasma sodium, **b** community-acquired vs. hospital-acquired hyponatraemia, **c** the Charlson comorbidity index, **d** serum albumin tertiles, **e** the absence vs. normalisation of plasma sodium and **f** the performing of urine analysis

In-hospital mortality for the entire cohort was 24.5 %. Twenty-five percent of the patients were deceased after 34 days and 50 % after 609 days, respectively. Mortality appeared lower in the group with the most severe hyponatraemia (Table 5 and Fig. 3) although did not reach statistical significance. In contrast, community-acquired severe hyponatraemia had a significantly better prognosis (Table 6 and Fig. 3). Among the 111 patients who survived their initial hospitalisation, 51.6 % were re-hospitalised for any cause and 11.3 % for severe hyponatraemia within 6 months. Patients who died during initial hospitalisation had a mean plasma sodium of 131.6 ± 11.2 mmol/l at time of death and 58.3 % were normonatraemic, values similar to those observed in survivors (mean plasma sodium 132.4 ± 6.5 mmol/l (*P* = .71) and 56.8 % normonatraemic (*P* = .99)).

**Table 6:** Characteristics and outcomes of patients with hospital-acquired hyponatraemia *versus* community-acquired severe hyponatraemia

	All patients (*n* = 147)	Hospital-acquired severe hyponatraemia (*n* = 52)	Community-acquired severe hyponatraemia (*n* = 95)	*P* ^*^
Plasma sodium (mmol/l)				
- on admission - lowest - at discharge - at discharge in survivors - Highest – lowest - Normalisation (%)	121.5 ± 10.4 114.9 ± 4.7 132.2 ± 7.9 132.9 ± 5.9 20.8 ± 8.6 84 (57.1)	133.1 ± 5.7 116.4 ± 3.9 130.3 ± 8.8 133.2 ± 6.9 20.0 ± 6.9 25 (48.1)	115.2 ± 6.0 114.1 ± 4.9 133.2 ± 7.2 132.7 ± 5.6 21.3 ± 9.4 59 (70.2)	<.001 <.001 .07 .84 .39 .12
Severe neurologic symptoms (%)	13 (8.8)	4 (7.7)	9 (9.5)	.99
Charlson comorbidity index	3.8 ± 3.0	4.92 ± 3.4	3,3 ± 2,6	.005
Potential causes of hyponatraemia	2.9 ± 1.3	3.5 ± 1.3	2.6 ± 1.3	<.001
Diagnosis				
Unknown (%)	69 (46.9)	30 (57,7)	39 (41.1)	.06
Potomania (%)	8 (5.4)	1 (1,9)	7 (7,4)	.26
Heart failure (%)	13 (8.8)	4 (7,7)	9 (9,5)	.99
Cirrhosis (%)	4 (2.7)	0 (0)	4 (4.2)	.30
Diuretics (%)	6 (4.1)	1 (1,9)	5 (5,3)	.42
Hypotonic fluids (%)	3 (2.0)	3 (5,8)	0 (0)	.04
Dehydration (%)	22 (15.0)	7 (13,5)	15 (15,8)	.81
CSWS (%)	2 (1.4)	1 (1.9)	1 (1.1)	.99
SIADH (%)	28 (19.1)	6 (11,5)	22 (23,2)	.12
Hypothyroidism (%)	3 (2.0)	0 (0)	3 (3.2)	.55
Adrenal insufficiency (%)	4 (2.7)	3 (5.8)	1 (1.1)	.13
Number of diagnoses reported per patient	0.65 ± 0.7	0.52 ± 0.75	0.72 ± 0.69	.05
Urine analysis (%)	109 (74.2)	39 (75.0)	70 (73.7)	.99
- Sodium (%) - Osmolality (%) - Urea (%) - Uric Acid (%)	109 (74.2) 38 (25.9) 102 (69.4) 17 (11.6)	39 (75.0) 12 (23.1) 37 (71.2) 5 (9.6)	70 (73.7) 26 (27.4) 65 (68.4) 12 (12.6)	.99 .69 .85 .79
Death during hospitalisation (%)	36 (24.5)	21 (40.4)	15 (15.8)	.001
Death during follow-up (*n* = 139) (%)	74 (50.3)	35 (70.0)	38 (42.7)	.003

*CSWS* Cerebral Salt Wasting Syndrome, *SIADH* Syndrome of Inappropriate Antidiuretic Hormone secretion, ^*^between hospital-acquired and community-acquired severe hyponatraemia

In univariate Cox analysis (Table 7), mortality was significantly and positively associated with female gender, onset of severe hyponatraemia during hospitalisation, persistence of hyponatraemia, absence of diagnosis, number of potential causes, lack of specific treatment for hyponatraemia, Charlson index and hypoalbuminaemia. Conversely, dehydration was associated with better prognosis.

**Table 7:** Uni- and multivariate Cox survival analysis

	Univariate Cox analysis HR [95CI]	*P*	Multivariate Cox analysis^a^ HR [95CI]	*P*
Male vs. Female	0.57 [0.36–0.90]	.02	0.69 [0.40–1.20]	.19
Age	0.99 [0.97–1.01]	.37		
Hospital vs. community-acquired hyponatraemia	2.24 [1.41–3.56]	.001	1.23 [0.67–2.27]	.51
Chronic vs. acute hyponatraemia	0.72 [0.28–1.85]	.50		
Symptomatic hyponatraemia	0.80 [0.51–1.27]	.36		
Severe neurologic symptoms	0.85 [0.37–1.97]	.71		
Nadir of plasma sodium	1.02 [0.97–1.08]	.35	0.95 [0.89–1.01]	.13
Urine analysis	0.64 [0.39–1.06]	.08	0.48 [0.27–0.85]	.01
Absence of diagnosis	1.77 [1.11–2.81]	.02	1.07 [0.55–2.07]	.84
Number of potential causes	1.45 [1.23–1.70]	<.001	1.07 [0.84–1.35]	.59
Treatment for hyponatraemia	0.34 [0.19–0.60]	<.001	0.67 [0.30–1.52]	.34
Accuracy of treatment	0.95 [0.43–2.08]	.90		
Plasma sodium normalisation	0.61 [0.39–0.97]	.04	0.35 [0.20–0.62]	<.001
Excessive correction	0.64 [0.33–1.25]	.19	0.76 [0.33–1.77]	.52
Charlson comorbidity index	1.28 [1.19–1.38]	<.001	1.23 [1.13–1.34]	<.001
Serum albumin (g/l)	0.91 [0.88–0.95]	<.001	0.88 [0.84–0.92]	<.001

*HR* Hazard ratio, 95CI: 95 % confidence interval. ^a^The multivariate model included the clinical relevant parameters and those with a *P*-value less than 0.2 in univariate analysis

In multivariate analysis (Table 7), survival remained associated with the normalisation of plasma sodium (HR 0.35; *P* = .001) and parameters associated with comorbidities, Charlson index (HR 1.23; *P* < .001) and serum albumin (HR 0.88; *P* < .001). Moreover, the prescription of a urine analysis was highly associated with good outcome (HR 0.48, *P* = .01). There was no statistical interaction between these factors. Association of these factors with mortality is illustrated in Fig. 2. Although the survival curves separated early, the normalisation of plasma sodium was not associated with in-hospital mortality (25 % upon normalisation vs. 23.8 % if persistent hyponatraemia, *P* = .99) whereas it was associated with mortality at the end of follow-up (42.9 % upon normalisation vs. 60.3 % if persistent hyponatraemia at hospital discharge; *P* = .046).

## Discussion

Severe hyponatraemia below 120 mmol/l is a serious condition and is associated with hospital mortality in about one quarter of patients, in accordance with previous studies [10–13].

The contribution of hyponatraemia in the death of affected patients remains nonetheless a matter of debate. Indeed, the most recent studies suggest that patients are more prone to die from their comorbidities than from hyponatraemia per se [14, 23]. In our multivariate analysis, patients in whom plasma sodium was normalised were found to have a significantly better survival (HR 0.35 [0.20–0.62]), independently of underlying comorbidities (Charlson comorbidity index and serum albumin). Moreover, patients with successful normalisation of their plasma sodium had a similar Charlson index than patients who did not. The absence of relationship between normalisation of plasma sodium and in-hospital mortality suggests that the absence of normalisation was not due to premature in-hospital mortality, prior to normalisation of plasma sodium, but rather that subsequent mortality may be due to the absence of plasma sodium normalisation. However, nearly 60 % of patients who died during hospitalisation had normalised their plasma sodium. In addition, mortality was highly associated with the Charlson index and with hypoalbuminaemia, thereby suggesting that comorbidities and hyponatraemia may act synergistically to increase the risk of death in these patients.

While some studies have reported an association between the magnitude of hyponatraemia and mortality [5, 11, 24], we were unable to draw similar conclusions. The study by Mohan et al. regarding at-home patients and who were probably asymptomatic, found a clear positive correlation between the severity of hyponatraemia and mortality [24]. In the study conducted by Gill et al. in hospitalised patients, mortality was positively correlated with the lowest plasma sodium during hospital stay but not with the plasma sodium measured on admission, which was, on the contrary, inversely correlated with mortality [11]. In this latter study, mortality was also associated with the decline in plasma sodium during hospitalisation. These data suggest that mortality was more related to the cause of hyponatraemia, to hospital-acquired hyponatraemia or to the inadequate management of hyponatraemia than to hyponatraemia per se. A recent Danish study including hospitalised patients is in accordance with our findings, in which the authors found that mortality was not increased when serum sodium decreased below 132 mmol/l [25]. In contrast, there was even a trend toward better survival among patients with the most severe hyponatraemia. We hypothesise that these paradoxical findings observed in hospitalised patients are explained by the fewer comorbidities and the more severe neurologic symptoms associated with the more severe cases of hyponatraemia. Indeed, although not statistically significant because of the small sample size, patients herein with the lowest plasma sodium had less cardiovascular, pulmonary or renal comorbidities, all known to deeply impact mortality. The patients with the lowest plasma sodium were probably more easily detected as a metabolic emergency and thus more aggressively treated due to more apparent symptomatic presentation specifically related to hyponatraemia. Accordingly, aetiological workup identified by the prescription of urine analysis, specific treatment for hyponatraemia such as fluid restriction, hypertonic saline or demeclocycline, as well as the presence of an etiological diagnosis were more frequent in the more severe group.

The Charlson comorbidity index is a well-known prognostic score validated in a multitude of diseases including severe hyponatraemia [23, 26]. However, despite numerous demonstrations that hypoalbuminaemia is correlated with mortality in both renal and non-renal diseases, such prognostic link has yet to be described in hyponatraemic patients [27–32]. Serum albumin can be considered as a supplemental marker of severity, independent of the Charlson index, which does not take into account either the nutritional status or inflammation.

Slightly more than half of our severe hyponatraemic patients were symptomatic, in agreement with previous studies [10, 13, 16], while severe neurologic symptoms, such as seizures and coma, were significantly more frequent when plasma sodium was below 110 mmol/l, in agreement with prior pathophysiological data and retrospective observations [33, 34]. In the present study, the incidence of severe neurologic symptoms was not associated with the timing of the hyponatraemia, i.e., acute or chronic, although timing could not be determined in over half of the patients because of the unavailability of prior plasma sodium values. On the other hand, severe neurologic symptoms are more prone to occur in very acute hyponatraemia, i.e., developing in less than 24 h, whereas in the present study, acute hyponatraemia was defined as developing in less than 48 h, as recommended by current guidelines [35, 36].

As previously reported by others, severe hyponatraemia was more frequently community-acquired than hospital-acquired [10, 16]. As reported by others, severe hyponatraemia present at admission had a much better prognosis than hospital-acquired hyponatraemia despite lower initial plasma sodium [6]. In our study, this was likely the consequence of less comorbidity and possibly better care as suggested by a more frequent aetiological diagnosis.

The acute setting of hyponatraemia was not associated with either the presence of symptoms or with in-hospital mortality. However, this setting could not be determined in over 50 % of cases herein, typically due to lack of prior record, which limits further generalisation. This observation does illustrate, however, the difficulties in implementing the previous guidelines and why current guidelines recommend that treatment be based firstly on the presence of severe or moderately severe symptoms and secondly on the timing of setting in asymptomatic cases [36]. These guidelines also recommend considering hyponatraemia as chronic whenever the timing cannot be determined.

Contrary to the study of Kang et al. [23], but in agreement with other studies [16, 19, 37], we did not find a relationship between excessive rate of correction and mortality. Eighteen per cent of patients showed a rate of correction now viewed as excessive, a figure comparable to that of other studies [12, 13, 17]. This however does not mean overcoming the current limits of correction rate, since osmotic demyelination syndrome may occur in a delayed manner and without necessarily incurring death. In fact, it is the concern of this complication which has led, over time, to progressively reduce the rate of correction to those actually recommended: <10 mmol/l within the first 24 h and <8 mmol/l in any 24 h thereafter [36].

In our series, global care for hyponatraemia was often deemed insufficient. In nearly half of the cases, no aetiological diagnosis was mentioned, hence suggesting inadequate treatment. The frequent inadequate management of hyponatraemia, in particular for SIADH diagnosis, is a well-established observation which has recently been confirmed in the HN registry [20]. As already described by Huda et al., we also found that cases of severe hyponatraemia without aetiological diagnosis exhibited a 77 % excess mortality in univariate analysis although this excess was not significant in multivariate analysis [18]. Such lack of aetiological diagnosis may be related to insufficient or inadequate biological workup [18, 19]. Indeed, although measurement of plasma osmolality is recommended for initial assessment of any hyponatraemia, such measurement was performed in only 34 % of patients in the present series [36]. Nonetheless, measurement of plasma osmolality is recommended for excluding hyper- or isotonic hyponatraemia which is quite unlikely with a plasma sodium below 120 mmol/l [38]. Aetiological diagnosis may thus be better guided by measurement of urine electrolytes which was prescribed in 75 % of patients who, as expected, had a significantly lower mortality rate in multivariate analysis. The better outcome in patients in whom adequate laboratory tests are performed could be explained by higher success rates in correcting hyponatraemia as suggested by Verbalis et al. in the HN registry; however this was not the case in the study herein [39].

We also found a low percentage of untreated patients in the present study. This discrepancy with the frequent absence of aetiological diagnosis is likely due to the retrospective nature of the study, since any therapeutic change following the onset of severe hyponatraemia was considered as treatment of this disorder. However, fluid restriction was prescribed in only two thirds of patients. The lack of treatment for severe hyponatraemia may be related to the more numerous comorbidities and an overall prognosis deemed too pejorative by the medical staff in charge of the patient’s care. Accordingly, 80 % of untreated patients died during follow-up.

## Strengths and limitations

Our study has certain limitations. First, the retrospective nature imposing the study of handwritten medical records certainly limited the access to certain valuable information, including final diagnosis. Secondly, plasma sodium was not corrected herein by plasma glucose as recommended, as it was difficult to retrospectively obtain blood glucose for each value of plasma sodium [38]. Nevertheless, the low threshold value chosen to characterise severe hyponatraemia makes it unlikely that hyperglycaemia could have completely distorted the relationship between plasma sodium and osmolality. In addition, two studies found identical mortality rates with and without using correction of osmolality by glucose [4, 14]. On the other hand, in the present series, the relatively limited number of patients from one single centre allowed access to the entire medical records and to correlate the prognosis with a wide variety of parameters. Thus this is, to our knowledge, the first study demonstrating that prognosis is influenced independently by hyponatraemia itself, by comorbidity, and by medical management.

## Conclusion

Severe hyponatraemia is a serious condition associated with a very high mortality rate. Nearly two thirds of patients died with normal plasma sodium indicating that patients are more prone to die from the cause of hyponatraemia and/or comorbidities than from hyponatraemia per se. However the absence of normalisation in plasma sodium also increases the risk of death. Clinical management plays a crucial role as highlighted by the fact that lack of initial urine analysis as well as absence of both aetiological diagnosis and normalisation of plasma sodium were each associated with increased mortality. Improving the prognosis of these patients may rely on a careful management of all associated comorbidities, including improving the training of medical personnel involved in the care of hyponatraemic patients.

## Abbreviations

BMI: Body mass index
BNP: Brain natriuretic peptide
CSWS: Cerebral salt wasting syndrome
eGFR: Estimated glomerular filtration rate
HR: Hazard ratio
SIADH: Syndrome of inappropriate anti-diuretic hormone secretion
TSH: Thyroid-stimulating hormone
